# Insights From a Clinically Orientated Workshop on Health Care Cybersecurity and Medical Technology: Observational Study and Thematic Analysis

**DOI:** 10.2196/50505

**Published:** 2024-07-11

**Authors:** Isabel Straw, Irina Brass, Andrew Mkwashi, Inika Charles, Amelie Soares, Caroline Steer

**Affiliations:** 1 Institute of Health Informatics University College London London United Kingdom; 2 Department of Science, Technology, Engineering and Public Policy University College London London United Kingdom; 3 Newcastle University Newcastle United Kingdom; 4 University College London London United Kingdom

**Keywords:** digital health, clinical medicine, biotechnology, medical device, device regulation, medical education, eHealth, digital medicine, health care, health care cybersecurity, internet of medical things

## Abstract

**Background:**

Health care professionals receive little training on the digital technologies that their patients rely on. Consequently, practitioners may face significant barriers when providing care to patients experiencing digitally mediated harms (eg, medical device failures and cybersecurity exploits). Here, we explore the impact of technological failures in clinical terms.

**Objective:**

Our study explored the key challenges faced by frontline health care workers during digital events, identified gaps in clinical training and guidance, and proposes a set of recommendations for improving digital clinical practice.

**Methods:**

A qualitative study involving a 1-day workshop of 52 participants, internationally attended, with multistakeholder participation. Participants engaged in table-top exercises and group discussions focused on medical scenarios complicated by technology (eg, malfunctioning ventilators and malicious hacks on health care apps). Extensive notes from 5 scribes were retrospectively analyzed and a thematic analysis was performed to extract and synthesize data.

**Results:**

Clinicians reported novel forms of harm related to technology (eg, geofencing in domestic violence and errors related to interconnected fetal monitoring systems) and barriers impeding adverse event reporting (eg, time constraints and postmortem device disposal). Challenges to providing effective patient care included a lack of clinical suspicion of device failures, unfamiliarity with equipment, and an absence of digitally tailored clinical protocols. Participants agreed that cyberattacks should be classified as major incidents, with the repurposing of existing crisis resources. Treatment of patients was determined by the role technology played in clinical management, such that those reliant on potentially compromised laboratory or radiological facilities were prioritized.

**Conclusions:**

Here, we have framed digital events through a clinical lens, described in terms of their end-point impact on the patient. In doing so, we have developed a series of recommendations for ensuring responses to digital events are tailored to clinical needs and center patient care.

## Introduction

The patient who seeks medical care due to a medical device fault, cybersecurity exploit, or failure in digital health infrastructure may encounter a clinical team that lacks an understanding of the nature of their condition [1-7]. These digital events are often framed as computing issues, yet in practice they manifest as patient symptoms and signs and pose significant challenges to the treating clinicians at the point-of-care [1-9]. In our digitized society, where health care provision is increasingly reliant on technological infrastructure, the “Internet of Medical Things,” and connected and intelligent medical devices, computing issues are increasingly translating into clinical complaints [2-8,10-16].

### The Landscape of Digital Health Technologies

The proliferation of digital technologies in the health care sector has accelerated over the past decade, with new medical devices entering the market, the growth of consumer health technologies, and the introduction of novel digital tools to hospital workflows (eg, cloud-connected care platforms, digital assistants, and remote monitoring) [10-12,17-22]. These devices are connected to communication networks and the Internet to send, store, and process data in the cloud, forming integral components of the evolving “Internet of Medical Things” [10-12,21,23]. A subset of medical devices comprise stand-alone software, known as Software as a Medical Device, which may incorporate varying degrees of artificial intelligence (AI) approaches and locked or adaptive machine learning [10,17,24]. These novel digital tools present many opportunities for improving patient care, yet they have also introduced new vulnerabilities into the health care system that may impact patient safety [10,16,21,24-28].

### New Risks in the Digital Health Landscape

Our increased reliance on digital infrastructure opens us up to new digital risks, exemplified by the increasing number of cyberattacks affecting the health care sector [2,25-28]. Borycki et al [29] have provided an overview of the new types of technology-induced errors that have arisen in the health care system with the introduction of health information technologies. The authors detailed the dangers of an overreliance on technology and explored the specific challenges faced by clinicians who are “digital natives,” including unrealistic expectations towards fault tolerance and the availability of digital systems [29,30]. Sax et al [30] consider the potential patient harm associated with a range of IT failures, including loss of system availability, loss of data, and loss of data integrity. Alemzadeh et al [16] place technological events in their clinical context, linking adverse computer incidents to end-point clinical symptoms, uncovering a range of safety-critical computer failures that have resulted in significant patient harm and death.

In the United Kingdom, the retrospective analyses of the National Health Service (NHS) WannaCry attack highlighted the importance of cybersecurity for patient safety; yet, many NHS hospitals still lack guidance regarding the incident response to a cyberattack [28]. Furthermore, security researchers have sounded the alarm regarding the potential for individual-level health care attacks termed “MedJacking,” referring to the remote manipulation of patients’ medical devices such as insulin pumps and deep brain stimulators [6,31]. When such adverse events occur, research has described the challenges encountered by clinicians for whom the clinical manifestations of technological failures may be unfamiliar [1,5,15]. A recent review of patient illnesses stemming from digital technologies—termed “Biotechnological syndromes”—detailed a range of clinical presentations relating to implanted medical devices (eg, complications of neurostimulators) and technologies within the wider health care system (eg, harms from failures in drug delivery systems) [5].

Clinical complications may also arise from digital technologies not traditionally thought of as “digital health” technologies. In particular, the rise in biohacking technologies has presented unexpected challenges to clinicians, as described by Fram et al [7] and Gangadharbatla [32] in their review of the clinical considerations of consumer implants and microchips. Clinical presentations may also be affected by technology through unexpected mechanisms, as highlighted in cases of technology-facilitated abuse in health care settings [33,34]. Research from the domestic violence field has described the harms faced by patients encountering technology-facilitated abuse (eg, harm inflicted through the manipulation of smart devices) and the need for clinicians to update safeguarding protocols to encompass these risks [33,34].

The introduction of health care AI has brought forward novel ethical questions regarding accountability and liability, with researchers raising concerns about the removal of the “human-in-the-loop” in clinical systems that embed autonomous functions [10,26]. Farhud and Zokaei [35] and Habli et al [36] discuss the challenges of determining moral accountability in complex sociotechnical systems involving AI software, and Fahrud and Zokaei frame these challenges through the traditional pillars of medical ethics (autonomy, beneficence, nonmaleficence, and justice). Dufour et al [37] highlight the potential clinical dangers of autonomous systems in their report, which describes several cardiac arrests stemming from algorithmic errors in a series of ventilators. In addition to concerns surrounding AI autonomy, ethicists have illuminated further issues associated with these technologies, including the risks of bias and discrimination in AI-supported clinical decision tools [38-41].

### Clinical Medicine and Digital Health Risks

Currently, clinicians receive little training on the emerging digital technologies that their patients rely on for care and that professionals depend on to perform their work [1-8,15]. As detailed by Sally Adee in her comprehensive history of the life sciences and physical sciences, the separation of these 2 scientific domains over the past 200 years has resulted in distinct professional languages and expertise, which often struggle to understand one another [42]. However, with the advance of biodigital convergence and the growing prevalence of digital health technologies, clinicians are increasingly likely to encounter patients that are no longer purely biological but rely on varied digital devices requiring an understanding of the physical sciences [5,42,43]. Furthermore, the government bodies tasked with overseeing digital health technologies have faced the challenge of understanding biomedical innovations arriving from overseas, requiring an alignment of domestic standards with the global market [10,24,44]. As a result, the technologies that play a key role in shaping a patient’s journey may be poorly understood by the health care practitioners and the governmental agencies responsible for public oversight. ‬‬‬‬‬

### Research Aim

The disciplines of clinical medicine, cybersecurity, and engineering have long operated in silos, and as a result, we lack effective frameworks for patients whose health complaints emerge from the intersection of these domains. In this paper, we present the findings of a confidential multistakeholder workshop that bridged the gap between these professions, facilitating an interdisciplinary discussion on the key challenges affecting digitally dependent patients. We provide insights from frontline staff on the difficulties faced during digital events (eg, cyberattacks and device failures), detail differing perspectives from varied stakeholder groups, and present a series of recommendations for ensuring best clinical practice in the evolving digital health care infrastructure.

## Methods

### Overview

The workshop titled “Emerging Digital Technologies in Patient Care: Dealing with connected, intelligent medical device vulnerabilities and failures in the health care sector” took place at Goodenough College, London, United Kingdom in February 2023. The workshop was part of an EPSRC-funded project investigating how health care systems, regulations, and standards are responding to the cybersecurity and algorithmic integrity challenges posed by the growing use of connected and intelligent medical devices.

### Participant Recruitment

The workshop of 52 participants had representation from the European Union, the United Kingdom, and the United States, involving a wide range of stakeholder groups (Table 1).

All workshop participants were recruited based on their practical experience and expertise in digital health care. Limited snowball sampling was used to identify practitioners and experts in the field. Participants were recruited via email by the research team, leveraging the academic, clinical, and professional networks of IS, IB, and AM and their affiliated institutions. Thus, participants were aware of the researchers’ academic profiles prior to the workshop and were provided with detailed information in advance describing the goals of the research.

No patient or health care data were used for the workshop, and all scenarios (Textbox 1 [43]) were fictitious and informed by published research. Workshop discussions were held under the Chatham House Rule and neither the identity nor the organizational affiliation of participants will be disclosed in research outputs derived from the workshop. Participants were identified solely by broad stakeholder categories (Table 1) using different badge colors for note-taking purposes. No compensation was provided to workshop participants.

**Table 1:** Details of workshop participants and their disciplinary backgrounds (N=52).

Stakeholder category	Participants
Health care professionals or clinicians	20
Public body representatives	3
Device manufacturers and developers	6
Standards bodies representatives	4
Regulatory consultants and advisers	5
Academic professionals	14

**Details of the clinical scenarios designed for table-top exercises specific to different specialties, all of which are based on published case reports.**

**Acute medicine**

Caring for medical patients during a weekend cyberattack: In scenario 1, a hospital cyberattack compromised a (1) cloud-based platform that detailed chemotherapy regimens for oncology patients, (2) smart drug-delivery systems within the hospital, and (3) patient electronic health records (EHRs). The patients described within the scenario all required specific dosing of medications and careful fluid management; the group had to prioritize patients for care and design a wider hospital response.

**Acute medicine**

Managing unwell patients during a cyberattack on the acute medical unit (AMU): scenario 2 focused on clinical cases requiring careful management of acid-base conditions that necessitated effective blood gas analysis (diabetic ketoacidosis and renal failure) and attentive fluid management (decompensated heart failure); however, the laboratory, blood gas machine, and smart pumps were all compromised.

**Acute medicine**

Treating blind—patient care during a radiological cyberattack: scenario 3 focused on patient conditions where clinical decision-making relied on radiological information, including (1) identification of the pneumothorax for chest drain insertion, (2) use of a chest x-ray to confirm the position of the nasogastric tube, (3) magnetic resonance imaging to diagnose cauda equina syndrome, and (4) computed tomographic imaging to identify an intercranial bleed. In the scenario, the EHR system was unavailable, and the radiological imaging system was known to be compromised (although the impact on the integrity of scans was unclear).

**Surgery and obstetrics**

Mother, baby, and spinal cord stimulator: In scenario 4, the team needed to decide on the best management for a pregnant patient presenting with signs of labor, who reported having a closed-loop spinal cord stimulator in situ. The patient is likely to need a caesarean section due to the breech position of the baby but has not had a preanesthetic evaluation, and there is no available information regarding the spinal cord stimulator. The model of a spinal stimulator has artificial intelligence–integrated functionalities, including the self-adjustment of settings based on patient posture. The team must design a safe clinical care plan that accounts for the device and any complications.

**Emergency and intensive care**

Patient care and ventilator autonomy: scenario 5 was based on the intensive care unit, where a ventilator malfunction causes patients to go into cardiac arrest. The ventilators have integrated automated functions that allow them to update their own settings; however, the attending clinicians are unable to interpret the settings of the machine. The team was tasked with determining the immediate clinical response and discussing the wider implications of closed-loop life support systems in critical medical settings.

**General practice**

Seizure outbreaks in epilepsy management apps: In scenario 6, a teenager presents to her general practitioner with a relapse in seizure symptoms, having a known diagnosis of epilepsy that had been previously well controlled. It is suggested that the epilepsy management app on her phone has been compromised. The team was tasked with planning an appropriate response for this patient and the wider population’s health implications.

### Ethical Considerations

Ethical approval was obtained from the UCL Research Ethics Committee (no 222137/001-0023A). A participant information sheet detailing the research project and a consent form were circulated in advance, and participants were asked to return the consent form via email. All participants provided written informed consent for their contributions to be used as anonymized research data.

### Workshop Structure

The workshop consisted of two parts: (1) a series of expert talks followed by a Question and Answer (Q&A) session and group discussion, and (2) breakout table-top exercises in which participants discussed and designed a response to a clinical scenario complicated by technology (Textbox 1). Clinical scenarios were written to account for the diversity of health care technologies and the disciplinary backgrounds represented in the room. All scenarios were based on published case reports and reported issues related to digital health technologies. The full case scenarios are provided in Multimedia Appendix 1 and detailed in the web-based workshop description [45]. Notetakers were present throughout the workshop to record details of Q&A sessions, group discussions, and table-top exercises (audio and visual recording was not used). Notetakers received in-person training in advance of the workshop to review the research materials, discuss the scenarios, address any questions, and agree on a framework for the data collection process.

### Research Data Analysis

Extensive notes taken during the workshop were retrospectively analyzed and the data from the 5 note-takers was cross-referenced to ensure consistency in the reported results. Four of the researchers coded the qualitative data and undertook an inductive thematic analysis to extract major and minor themes present within the text. Anonymized examples of clinical cases involving technology provided by clinicians were collected as use cases and described in the results.

## Results

Our results are divided into themes extracted from panel Q&As and group discussions, and those identified from the breakout exercises on different clinical scenarios.

### Group Discussions: Identified Themes

#### Digital infrastructure: “The Hospital Still Has Windows 10”

The outdated IT infrastructure of the NHS in the United Kingdom is a known risk for cyberattacks, as these systems can more easily be exploited by malicious actors [25-28,45,46]. Participants described the compounding effect that poor IT infrastructure has on their digital behavior, which may further exacerbate the risk of cyber-exploitation. For example, the fact that IT systems are often slow, broken, or unreliable results in clinicians sharing computers, logging in and engaging in risky cyber-hygiene practices. Additionally, the current pressure on NHS staff due to underfunding and staff shortages results in a lack of capacity for additional training on cybersecurity.

Clinicians expressed that it was hard to get excited about the introduction of digital systems when the basic health care needs of their patients were not being met. Further, practitioners shared concerns regarding the impact of digital innovation on health care inequalities, as access to newer technologies is often mediated by wealth therefore deepening socioeconomic disparities in health outcomes. We heard examples of tech-poverty affecting patients, including accessibility issues related to the rise of telehealth, which is inaccessible to disadvantaged patient groups. Clinicians also raised concerns regarding technology-facilitated abuse, with one participant describing the malicious use of GPS tracking technologies to “Geofence” young women and girls within specific city boroughs, such that abusive parties would receive notifications if they left defined geographic areas (Textbox 2).

**Anecdotes and examples of patient presentations related to technology that were disclosed by participants at the workshop.**

**Hospital medicine**

In a case of a malfunctioning piece of radiological equipment, we had a case where 3 dozen patients were exposed with higher doses, above the diagnostic reference level.

We know we have issues with pacemaker batteries, but we don’t know how many are affected. We need to understand how to manage that risk.

Women who was a 35-year-old diabetic patient who died and it was a surprise to the clinical team. The patient had been on a pump and the clinician discussed with the coroner if the pump could have contributed? The husband had thrown the insulin pump in the bin, no one had looked at it so there was no clear cause. We need to collect devices after a death, maybe give them to the police.

**Surgery**

In Obstetrics and Gynecology, there was a case of the wrong woman being given an Emergency C-Section because the communication lines from 2 fetal CTGs overlapped, and one baby’s readings had been assigned to the wrong mother.

**Community care**

In general practice surgery, Apple watches causing patients to think their heart rhythms are abnormal.

Seen issues in general practice with tech-abuse and GPS tracking. Young girls in some communities have to stay inside their borough, otherwise an alarm goes off to their family or partner.

#### Trust and Medical Devices: “The Device Should Be Considered Guilty Until Proven Otherwise”

Clinicians expressed concerns regarding the often-unchallenged assumption that exists in the medical community regarding device functionality, with one practitioner sharing the view that “99.9% of clinicians wouldn’t expect a device to fail,” reinforced by peers stating that they’d be highly unlikely to suspect embedded technology as a source of pathology. In cases where medical devices do fail, manufacturers shared that it is not always easy to identify the cause. Companies may be able to take a piece of technology back and attempt to replicate the failure mode; however, often these faults are attributed to unknown internal or external factors.

Manufacturers highlighted the risk of eroding patient trust when device malfunctions are poorly communicated, detailing the historic challenges that they have faced when communicating issues to patients, stating, “It is easy to spend millions on recalls when it is not necessary.” Workshop participants discussed that manufacturers are often cognizant that technologies fail but may feel that publicizing all of these events could cause unnecessary alarm, especially in cases where a fault (eg, a software bug) is likely inconsequential but causes fear due to its presence in a consequential technology (eg, a ventilator). Lastly, representatives from the policy domain highlighted the potential for fake news and disinformation that could result from misinterpreted reports of technological failures. Participants also raised issues of public versus private interests, exploring the role that financial incentives play when making decisions regarding device fault disclosures.

#### Responsibility and Liability: “Clinicians Don’t Have Time to Report This”

Views differed between clinicians and manufacturers on the topics of device failure reporting, post-market surveillance, and professional liability in cases of patient harm. Clinicians shared frustrations that they “don’t have time to report this” (referring to device malfunctions), stating that responsibility for consistent surveillance of deployed devices should be with the manufacturer. The issue of transparency was evident when discussing the communication between clinicians, manufacturers, and regulatory agencies, as manufacturers often found themselves limited in the information they could share due to commercial confidentiality. Furthermore, there was confusion as to whether reporting device errors was mandatory, with clinicians stating that this was a voluntary (and unfortunately often underperformed) action, and other participants stating this was an obligation.

Workshop participants heard from representatives from the consumer implant industry, who described the increasing uptake of implanted radio frequency identification and near-field communication chips in younger generations. Such body modification technologies that are sited under the skin can cause medical complications (eg, infections, soft tissue injury); however, these technologies do not fall under medical device regulation. It manifested that members of the public occasionally approach these companies, expressing fears of being chipped by “the government” or “aliens”; yet, in the case of potentially serious mental health concerns, these individuals are not redirected to appropriate health care support. Additional ethically contentious cases were discussed, including requests from family members to “chip” older relatives with dementia, or younger children. There was a clearly identified need for improving safeguarding referral pathways.

### Clinical Scenarios

The focused disciplinary table-top exercises facilitated a deeper dive into the specific clinical issues that may arise within each clinical domain.

#### Medical Scenarios—“With Most Tech, we Don’t Know How it Works, so we Don’t Know How to Trust it”

The 3 hospital-based medical scenarios described failures in cloud-based treatment platforms, laboratory equipment, radiological systems, and electronic health records. Clinicians initially drew parallels to previous experiences where IT had been compromised, referencing the WannaCry 2017 attack and climate events (eg, heat waves) affecting computer systems. Practitioners described the chaos of shifting to paper-based prescribing and note-taking during on-call and overnight hours, during which time they noted a lack of leadership and defined protocols for responding to the event clinically.

In designing their response, all groups reached consensus that the cyberattack should be classified as a “major incident,” activating a chain of responses including the recruitment of additional staff, awareness at the national level, communication across sites, an effective PR response, and the allocation of roles to those with sufficient skills and seniority. Senior team members suggested the development of battle bags and action cards that are commonly used in other major incident events, citing Grenfell Tower and the Manchester Bombing as examples. Clinicians demonstrated some awareness of available resources, including NHS Digital and Chief Information Officers; however, these resources were relatively unheard of within the groups.

The scenario suggested there were cybersecurity vulnerabilities in the drug delivery and laboratory systems present on the wards, to which health care staff reported a preference for shutting down technology entirely as a safety measure (while acknowledging that this could be unnecessary and cause delays and even more harm). When discussing plans to turn off digital systems, the groups shared their concerns about what disabling devices would do and whether there was a default safe mode that could protect patients. In shutting down digital equipment, participants decided to return to rudimentary clinical techniques, including dripping (the act of delivering intravenous medications based on drips of liquid). The teams raised concerns for younger generations of clinicians who may not have this nondigital foundation to fall back on.

Lastly, practitioners identified the patients most at risk of harm in the context of IT manipulation in the hospital and developed a clinical hierarchy specific to digital threats. The patients with diabetic ketoacidosis and cauda equina were identified as likely to experience the worst outcomes due to their reliance on laboratory and radiological resources for treatment. By prioritizing the patients according to the role that technology played in their clinical management, participants framed the cyberattacks in clinical terms.

#### Surgical Scenario—“Mother, Baby, and Spinal Cord Stimulator”—“There Is no Way of Knowing How the Body Is Communicating With the Device”

The surgical group was tasked with managing a patient in active labor who had a potentially compromised implanted spinal cord stimulator. Through the discussion, it became apparent that there was a lack of clinical knowledge regarding the implications of the technology, and the health care staff opted to focus on the medical management that they did understand while putting the technical components to one side. In discussing the follow-up to the case, standard body representatives raised concerns that the incident would not be flagged as an adverse outcome, supported by clinicians who indicated that this would be unlikely to be reported.

#### Emergency and Intensive Care Scenario—“Patient Care and Ventilator Autonomy”

The clinicians first drew comparisons to historic crises events, including the 1952 Copenhagen crisis, in which medical students were recruited to manually ventilate patients [47]. When discussing the initial response to the scenario, the team discussed disconnecting all patients from the ventilators while acknowledging the challenges of doing this in an intensive care unit where patients are dependent on life support systems. The group discussed the specific implications of AI and closed-loop systems within medical equipment, identifying the central issue of trust and a lack of understanding of the technological mechanisms. The lack of training in AI systems was felt to be compounded by the absence of training regarding cyberattacks, with clinicians sharing that they had “never had a training day on what would happen in this scenario.”

#### Community Care and General Practice Scenario—“Seizure Outbreaks in Epilepsy Management Apps”

The scenario based on community care described a teenage patient who experienced a relapse in seizure symptoms, suspected to be related to a malicious hack on an epilepsy app. The group began by identifying possible adverse health effects, including seizures, headaches, distress, loss of vision, loss of focus, visual effects, and airway compromise. At-risk patient groups were noted, including dementia patients and those with neurodiversity. For immediate clinical management, practitioners felt that staff were likely to tell the patient to avoid the app or their phone entirely, reiterating a theme heard in the other groups of completely disconnecting from the technology.

In contrast to the other groups, the general practitioners discussed reporting the case to the police and ministry of defense due to the concern of a malicious attack and the implications this could have for a large number of people using the app. Participants also described the need to involve parents as this was a pediatric case and the role of the app company in protecting their users.

### Uncovered Examples

During the workshop, practitioners provided anonymized anecdotes of patient harm where technology played a role. Textbox 2 provides these example cases. In reflecting on a patient death, one participant discussed the lack of postmortem guidance, highlighting that the disposal of devices as medical waste precludes the evaluation of their involvement in a death.

## Discussion

### Principal Findings

In a patient journey, the individual is likely to encounter various forms of technology, from their electronic health record, to advanced, interconnected, automated, and intelligent health care technologies [15,45,48,49]. In these journeys, health care staff are the immediate point of contact when clinical care goes wrong, and patient health deteriorates [1-5]. In this paper, we have explored the points of digital vulnerability that may contribute to patient illness along these trajectories and discussed the issues of cybersecurity, device failures, and faulty AI systems from the perspective of treating clinicians.

Clinicians from a diverse range of specialties responded in a similar manner when confronted with failures in digital devices, opting to immediately shut down the technology. Whether this was disconnecting patients from ventilators, disabling all medication smart pumps, or advising patients to turn off phones that were vulnerable to malicious hacks, the safest measure was often considered to be preventing any ongoing interaction between the technology and the patient’s physicality. The response is understandable given that clinicians receive little education on these tools and do not trust the systems or have the confidence to appropriately evaluate them.

Yet when clinicians are not informed of a potential technological failure, the default position appears to be the opposite and to trust devices entirely, such that “99.9% of clinicians wouldn’t expect a device to fail.” Hence, an interesting dichotomy exists—when a clinician has not been given reason to doubt a device, they will often trust the device over the patient (eg, believing the patient’s blood glucose data, as opposed to their subjective symptoms); however, once doubt is introduced, the clinicians opt to disregard the technology completely. The polarity of these reactions mirrors the black-and-white nature of the black-box technology that remains opaque to clinicians using the digital tools. This delicate relationship reinforces the importance of effective communication around medical device failures with clinicians as well as with the public.

The lack of reporting regarding digital adverse events is a regulatory and public policy concern. Textbox 2 provides a list of events we collected in this workshop, of which several remained unreported and describe significant patient harm. Within these stories, we heard new examples of biotechnological syndromes and forms of technology-facilitated abuse that add to the existing literature reporting issues of technology in domestic violence and the risks posed to vulnerable patients [5,33,34].

We heard differing opinions on where responsibility and professional liability should lie when patient harm occurs because of a technology malfunction, with clinicians sharing the view that manufacturers are responsible for ongoing follow-up and manufacturers stating that clinicians are responsible for the outcomes of patients with embedded devices. Health care professionals advocated for a higher standard to be placed on manufacturers with regard to patient trust and transparency, citing parallels to the Hippocratic oath and fundamental medical ethics taught in medical school. These suggestions have previously been made by several health care cybersecurity researchers who developed a “Hippocratic Oath for Connected Medical Devices” [39-41].

### Conclusions

Our research has taken the unique approach of positioning digital health care technology failures in their medical context, viewed through the lens of the clinician at the point of care. In doing this, we demonstrate how health care staff can form tailored clinical hierarchies when faced with health care cyberattacks, such as prioritizing patients’ dependence on digitally vulnerable systems (eg, spinal injuries requiring radiological imaging) or identifying at-risk groups of mobile screen-based hacks (eg, patients with epilepsy, those who are neurodiverse, and those with dementia). Understanding cyberattacks as clinical attacks in this manner provides an opportunity to form the guidelines and major incident response protocols that our participants identified as urgent and lacking resources in hospital settings.

Our findings illustrate gaps in clinical knowledge regarding medical technology and a lack of confidence in managing these scenarios, which can only be addressed with improved clinical education and training. To ensure effective patient care in our environment of evolving digital infrastructure, historic IT responses to cyberattacks and device failures must be married with the clinical needs and perspectives provided in this report. Our research shines a light on a critical and understudied area at the intersection of clinical medicine and digital health that requires greater research and professional guidance. We provide a series of recommendations based on our findings in Textbox 3.

**Recommendations.**

- **Hospital protocols and incident plans:**
  Cyberattacks should be treated as clinical attacks, framed similarly to other major incidents such as terrorist and extreme weather events [50]. To achieve this, cyberattack threat models should be developed with end-point clinical symptoms and signs in mind, necessitating input from clinicians and engineers. Hospitals and health care practices should develop major incident protocols that specify the clinical steps to be taken in a cyberattack, hierarchically prioritizing patient groups, and repurposing existing resources such as action cards, dedicated response teams, battle bags, and communication pathways for escalation.
- **Medical education and clinical training:**
  Health care practitioners require a fundamental understanding of the novel digital technologies that their patients rely on, in order to treat them effectively when digital complications arise. Software-based medical devices, especially those connected to communication networks and with AI-integrated functionalities, require continuous monitoring of their performance by hospital staff and their medical device inventory teams. Digitally themed professional courses through the UK Royal Colleges would incentivize an uptake of this training, in addition to integrating educational content into National Health Service Trust mandatory training modules, clinical orientation and induction weeks in hospitals, and medical school education curricula.
- **Academia and research:**
  Research focused on the health complications of digital technologies needs to advance at a parallel rate to the development and deployment of digital health care tools and devices. Future research focused on the symptoms and signs of digital failures and technological pathology would improve the ability of clinicians to diagnose these cases, and consequently report them to the appropriate bodies.
- **Manufacturer training and support:**
  An easily accessible interface between manufacturers and clinicians is required to ensure health care staff can find appropriate information about the performance of connected and intelligent medical devices in a timely manner. Manufacturers could develop “how-to” cards and clinically tailored resources about their digital medical devices, which would be more useful to health care staff than traditional user manuals.
- **Regulation and reporting:**
  Increased interaction is needed between regulatory agencies, such as the Medicines and Healthcare products Regulatory Agency, and clinical teams. Through events in medical schools, hospitals, and community health care practices, representatives of public and regulatory bodies may provide additional support to clinicians on the reporting processes and existing guidance regarding software-based and connected medical devices.

### Limitations

While we engaged a range of critical stakeholders in our workshop, we were limited by sample size and the representativeness of our participants. In future research, it would be beneficial to engage a wider range of clinical specialties, such as dermatologists, oncologists, radiologists, and neurologists, where digital health technologies are expanding rapidly. Furthermore, we have limited our focus to emerging digital technologies, with functionalities including telemetry, internet connectivity, and AI. As a result, we have not examined issues associated with static medical devices; for example, adverse reactions to materials used for hip implants.

## Appendix

Multimedia Appendix 1 Supplementary Material: Workshop Scenarios for Dealing with Connected, Intelligent Medical Device Vulnerabilities and Failures.

## Abbreviations

AI: artificial intelligence
NHS: National Health Service
Q&A: Question and Answer

## Data Availability

The data and clinical scenarios generated during and analyzed during this study are available in Multimedia Appendix 1.
