# Supplementary material for: Insights From a Clinically Orientated Workshop on Health Care Cybersecurity and Medical Technology: Observational Study and Thematic Analysis
Source: J Med Internet Res. 2024 Jul 11;26:e50505. doi: 10.2196/50505 (PMC11273071; doi:10.2196/50505)
Supplement: Multimedia Appendix 1 [file jmir_v26i1e50505_app1.docx]

**Supplementary Material: Workshop Scenarios for Dealing with Connected, Intelligent Medical Device Vulnerabilities and Failures**

Participants to part in six scenarios describing compromised or potentially compromised connected and/ or intelligent medical devices. The scenarios were fictitious but based on data collected from real-life cases of medical device malfunctions and cybersecurity incidents reported in the specialist literature.

**Scenario 1: Caring for medical patients during a weekend cyberattack**(Patients are referenced by age and sex, e.g., 17M = 17 year-old male)

It’s Saturday morning and you are a member of the general medical team. As part of the weekend team, you will be covering a range of medical specialties who are not on site during on call hours, including oncology and neurology. At the morning meeting you are informed by the hospital site manager that there has been a cyberattack overnight which has compromised the wireless network of the hospital, the computing systems, and some of the supporting online platforms. Now that the computing systems are down, you cannot access radiological or laboratory reports and do not have blood results for the patients. The hospital site manager also shares concerns regarding the current use of smart pumps on the wards, which have recently been demonstrated to have several cybersecurity vulnerabilities [1-5].

0930 – During the medical handover you are told about the following patients who require attention:

1. **Mr Chemo & Mrs Infusion:** Two patients on the oncology ward are due to have their chemotherapy over the weekend, however the online chemo-prescribing platform has been compromised and you do not have access to their previous records or lab results [6].
2. **Mr Pancreas:** Mr Pancreas was admitted overnight with severe vomiting, dehydration, and confusion, related to end-stage metastatic pancreatic cancer. He was started on the palliative care pathway, however the syringe drivers for palliative medication have not yet been prescribed.
3. **Mrs Sugar:** Mrs Sugar is a 21F with type 1 diabetes who was admitted with Diabetic Ketoacidosis (DKA) and started on an insulin infusion. She requires review due to increased confusion and vomiting.

Aside from the four patients handed over to you, there are 42 medical patients across the different wards that you are responsible for, who have a range of common medical problems including acute illness in the elderly, asthma exacerbations, chest infections, ischaemic heart disease, kidney injury and alcohol withdrawal.

**Case-specific questions**

1. How will you prioritise the care of these patients and how may this be influenced by the specific risks of the cyberattack?
2. How would you assess whether the systems and devices (e.g., smart pumps) you are using are safe and are behaving/ performing accordingly? Consider that a consequence of this cyberattack is that it is unclear which supporting platforms, devices, and systems have been compromised and which are working as expected.
3. If you are concerned about the safety and performance of these devices, when, and to whom, do you communicate these concerns?
4. Once IT indicates the systems are no longer vulnerable, what measures are taken (by you or the hospital) to ensure the devices you are using are safe and performing accordingly?
5. What concerns do you have about the reliability of the digital healthcare systems and devices you use or come in contact with? How do these concerns impact your clinical decision-making and professional responsibility?

**General questions**

1. What challenges do clinicians face when dealing with the consequences of a cyberattack resulting in compromised digital records, devices, machinery, and/ or systems?
2. For outpatient care where remote services (i.e. cloud-based services) are increasingly used to monitor and tailor treatment regimes, how would compromises to these critical, yet externally managed, services affect patient care?
3. What challenges do clinicians face when using medical software and/ or (quasi)autonomous systems in patient care?

**References**

[1] Pycroft, Laurie, and Tipu Z. Aziz. ‘Security of Implantable Medical Devices with Wireless Connections: The Dangers of Cyber-Attacks’. Expert Review of Medical Devices, vol. 15, no. 6, June 2018, pp. 403–06.

[2] Adashi, Eli Y., and Nicole M. Thomasian. ‘Medical Devices in Harm’s Way: Medjacking’. JAMA Health Forum, vol. 1, no. 1, Jan. 2020

[3] ‘Symbiq Infusion System: FDA Cybersecurity Warning’. Reactions Weekly, vol. 1564, no. 1, Aug. 2015, pp. 7–7. Springer Link, <https://doi.org/10.1007/s40278-015-4150-5>.

[4] ‘Smart Pumps in Practice: Survey Results Reveal Widespread Use, but Optimization Is Challenging’. Institute For Safe Medication Practices, 4 Apr. 2018.

[5] ‘New Report Highlights Challenges of Implementing “Smart” Pump Devices across NHS’. HSIB, 19 Mar. 2021, https://www.hsib.org.uk/news-and- events/new-report-highlights-challenges-of-implementing-smart-pump-devices-across-nhs/.

[6] Ades, Steven, et al. ‘Cancer Care in the Wake of a Cyberattack: How to Prepare and What to Expect’. JCO Oncology Practice, vol. 18, no. 1, Jan. 2022

[7] Faulds, Eileen R., et al. ‘Insulin Pump Malfunction During Hospitalization: Two Case Reports’. Diabetes Technology & Therapeutics, vol. 18, no. 6, June 2016, pp. 399–403. PubMed, <https://doi.org/10.1089/dia.2015.0434>.

[8] Warner, Lindsay, et al. ‘Malfunctioning Sufentanil Intrathecal Pain Pump: A Case Report’. Journal of Medical Case Reports, vol. 14, no. 1, Jan. 2020, p. 1. <https://doi.org/10.1186/s13256-019-2314-2>.

[9] Haase, Krystal K., et al. ‘Clinicians’ Experiences and Reflections from A Health System Cyberattack’. Jaccp: Journal Of The American College Of Clinical Pharmacy, vol. 4, no. 6, June 2021.

[10] Fields, Aaron M., et al. ‘Closed-Loop Systems for Drug Delivery’. Current Opinion in Anaesthesiology, vol. 21, no. 4, Aug. 2008, pp. 446–51. <https://doi.org/10.1097/ACO.0b013e3283007ecc>.

**Scenario 2: Managing unwell patients during a cyberattack on the Acute Medical Unit (AMU)**

**0800am:** You are a member of staff on the Acute Medical Unit (AMU) providing care for 28 patients with a range of medical conditions.

Overnight the following patients were admitted from the Emergency Department:

- 1. **Mr Sugar:** 85M with Hyperosmolar hyperglycemic state (HHS) on a background of type 2 diabetes, started on IV fluids and insulin due to significant ketonaemia [1-2].
  2. **Mr Lungs:** 62M with respiratory distress and confusion, diagnosed with an exacerbation of his underlying Chronic Obstructive Pulmonary Disease (COPD).
  3. **Mrs Heart:** 52F with shortness of breath, cough, and fever, on a background of Stage 2 Heart Failure.
  4. **Mr Kidney:** 68M with chronic kidney disease (CKD) admitted with shortness of breath, peripheral oedema and severe confusion.

On attempting to view the patient notes, you find that Cerner (the Electronic Patient Health Records System) will not open, and the ward manager informs you the hospital may be experiencing a cyberattack. As a result, you no longer have access to radiological imaging or blood test results. The ward manager also shares her concerns regarding the treatments being delivered to the ward patients via smart pumps (including IV medications, fluids, blood transfusions, and insulin) given the recent reports regarding malfunctions and cybersecurity vulnerabilities relating to these pumps [3-9].

Due to the laboratory results being unavailable, the healthcare assistant has been using the ward Arterial Blood Gas (ABG) machine to obtain rudimentary blood tests for each of your patients. Before attending to patients, your team review these results and you quickly notice these seem grossly abnormal with all patients being reported to have severe metabolic acidosis that is not in keeping with their clinical picture [8].

You must now begin your ward round of the 28 patients on the AMU, including reviewing the new patients admitted overnight.

**Case specific questions**

1. How will you prioritise the care of these patients and how may this be influenced by the specific risks of the cyberattack?
2. How would you assess whether the systems and devices (e.g. smart pumps) you are using are safe and are behaving/ performing accordingly? Consider that a consequence of this cyberattack is that it is unclear which supporting platforms, devices, and systems have been compromised and which are working as expected.
3. If you are concerned about the safety and performance of these devices, when and to whom do you communicate these concerns?
4. Once IT indicates the systems are no longer vulnerable, what measures are taken (by you or the hospital) to ensure the devices you are using are safe and performing accordingly?
5. What concerns do you have about the reliability of the digital healthcare systems and devices you use or come in contact with? How do these concerns impact your clinical decision-making and professional responsibility?

**General Questions**

1. What challenges do clinicians face when dealing with the consequences of a cyberattack resulting in compromised digital records, devices, machinery, and/ or systems?
2. For outpatient care where remote services (i.e. cloud-based services) are increasingly used to monitor and tailor treatment regimes, how would compromises to these critical, yet externally managed, services affect patient care?
3. What challenges do clinicians face when using medical software and/ or (quasi)autonomous systems in patient care?

**References**

[1] Gosmanov, Aidar R., et al. ‘Hyperglycemic Crises: Diabetic Ketoacidosis and Hyperglycemic Hyperosmolar State’. <http://www.ncbi.nlm.nih.gov/books/NBK279052/>

[2] NICE. Diabetic hyperglycaemic emergencies. Available from: [https://bnf.nice.org.uk/treatment-summaries/diabetic-hyperglycaemic- emergencies/](https://bnf.nice.org.uk/treatment-summaries/diabetic-hyperglycaemic-%20emergencies/)

[3] ‘Symbiq Infusion System: FDA Cybersecurity Warning’. Reactions Weekly, vol. 1564, no. 1, Aug. 2015, pp. 7–7. Springer Link, <https://doi.org/10.1007/s40278-015-4150-5>.

[4] ‘Smart Pumps in Practice: Survey Results Reveal Widespread Use, but Optimization Is Challenging’. Institute For Safe Medication Practices, 4 Apr. 2018.

[5] ‘New Report Highlights Challenges of Implementing “Smart” Pump Devices across NHS’. HSIB, 19 Mar. 2021, <https://www.hsib.org.uk/news-and-events/new-report-highlights-challenges-of-implementing-smart-pump-devices-across-nhs/>.

[6] Faulds, Eileen R., et al. ‘Insulin Pump Malfunction During Hospitalization: Two Case Reports’. Diabetes Technology & Therapeutics, vol. 18, no. 6, June 2016, pp. 399–403. PubMed, <https://doi.org/10.1089/dia.2015.0434>.

[7] Warner, Lindsay, et al. ‘Malfunctioning Sufentanil Intrathecal Pain Pump: A Case Report’. Journal of Medical Case Reports, vol. 14, no. 1, Jan. 2020, p. 1. PubMed, <https://doi.org/10.1186/s13256-019-2314-2>.

[8] Masayuki, et al. ‘Misdiagnosis of High Anion Gap Acidosis Owing to Instrument Error of a Device’. CEN Case Reports, vol. 8, no. 4, Nov. 2019, pp. 308–10. PubMed, <https://doi.org/10.1007/s13730-019-00413-4>

[9] FDA Report: Smiths Medical Recalls Certain Medfusion 3500 and 4000 Syringe Infusion Pumps for Software Issues That May Impact Infusion Delivery (2022).

**Scenario 3: Treating blind – Patient care during a radiological cyberattack**

**0830am:** You are a member of the medical team on the Acute Medical Unit (AMU) in a busy hospital and have just been informed that the hospital is facing a system-wide cyberattack which has compromised several NHS sites at once [1]. The hospital site manager informs you that the Electronic Health Records (EHR) system is unavailable, and that the IT team have advised against using PACS (the radiological imaging system in your trust) [2-4]. The radiology system is being investigated due to a technological failure that suggests that the images being displayed are inaccurate [5]. At this stage it is not clear whether the PACS system failures are linked to the cyberattack, to algorithmic malfunctions, or a combination of the two.

The following patients have been referred to you from the Emergency Department and for whom you do not currently have any investigation results:

1. **Miss Lung:** 24F admitted following a severe asthma attack requiring salbutamol and ipratropium nebulisers and IV magnesium sulphate. Her wheeze has resolved but she is now complaining of increasing left sided chest pain and shortness of breath.
2. **Mr Oesophagus:** 84M admitted following a stroke that resulted in left arm and leg weakness with severe dysphagia. He can no longer swallow and requires an NG tube for feeding and his regular medications (antihypertensives and antiepileptics).
3. **Mrs Legs:** 61F admitted with 6-hour history of lower back pain, bilateral burning leg pain and urinary incontinence, with a background of degenerative disc disease.
4. **Mr Brain:** 30M admitted following with a severe headache, pain when looking at lights, vomiting and drowsiness.

**Case specific questions**

1. How will you prioritise the care of these patients and how may this be influenced by the specific risks of the cyberattack and the radiological software not performing accordingly?
2. How would you assess whether the systems and devices (e.g. radiological imaging system) you are using are safe and are behaving/ performing accordingly? Consider that in this case, it is not clear why the PACS system is not performing accordingly.
3. If you are concerned about the safety and performance of these devices, when and to whom do you communicate these concerns?
4. Once IT indicates the systems are no longer vulnerable, what measures are taken (by you or the hospital) to ensure the devices you are using are safe and performing accordingly?
5. What concerns do you have about the reliability of the digital healthcare systems and devices you use or come in contact with? How do these concerns impact your clinical decision-making and professional responsibility?

**General questions**

1. What challenges do clinicians face when dealing with the consequences of a cyberattack resulting in compromised digital records, devices, machinery, and/ or systems?
2. For outpatient care where remote services (i.e. cloud-based services) are increasingly used to monitor and tailor treatment regimes, how would compromises to these critical, yet externally managed, services affect patient care?
3. What challenges do clinicians face when using medical software and/ or (quasi)autonomous systems in patient care?

**References**

[1] Ghafur, S., et al. ‘A Retrospective Impact Analysis of the WannaCry Cyberattack on the NHS’. Npj Digital Medicine, vol. 2, no. 1, Oct. 2019, pp. 1–7. www.nature.com, <https://doi.org/10.1038/s41746-019-0161-6>.

[2] Anderson T, Torreggiani W.C. ‘The Impact of the Cyberattack on Radiology Systems in Ireland’. Ir Med J; Vol 114; No. 5; P347. Available

at: <https://imj.ie/wp-content/uploads/2021/05/The-Impact-of-the-Cyberattack-on-Radiology-Systems-in-Ireland.pdf>

[3] Dameff, Christian, et al. ‘Cyber Disaster Medicine: A New Frontier for Emergency Medicine’. Annals of Emergency Medicine, vol. 75,no. 5, May 2020, pp. 642–47. ScienceDirect, <https://doi.org/10.1016/j.annemergmed.2019.11.011>.

[4] Eichelberg, Marco, et al. ‘Cybersecurity Challenges for PACS and Medical Imaging’. Academic Radiology, vol. 27, no. 8, Aug. 2020, pp. 1126–39. ScienceDirect, <https://doi.org/10.1016/j.acra.2020.03.026>.

[5] FDA (2022). Class 2 Device Recall ZAPX (Treatment Delivery). Available at: <https://www.accessdata.fda.gov/scripts/cdrh/cfdocs/cfRES/res.cfm?id=195555>

**Scenario 4: Mother, baby and spinal cord stimulator**

**Scenario description**

You are covering the Obstetrics ward during a weekend shift at a busy district general hospital (DGH). On arriving to the ward, the nursing team ask you to review a Ms Spine - a patient who has just arrived. At the same time the ward manager also informs you that the hospital may be experiencing a system-wide cyberattack, and as a result you do not have access to radiological imaging or laboratory tests [1-4].

Ms Spine is as 29F patient (Gravida 3, Para 2) with an intrauterine pregnancy at 38 weeks and 3 days' gestation who has presented to the labour and delivery unit in active labour. The patient quickly informs you of her past medical history, which includes a motor vehicle accident 2 years ago (occurring after the birth of her last child) that left the patient with lower back pain and bilateral neuralgias [5]. For these symptoms she underwent placement of a closed-loop spinal cord stimulator that auto-adjusts based on her posture. *(Participants can find further details on the spinal cord stimulator below).*

Aside from her back surgery, Ms Spine has a history of well-controlled asthma and her previous pregnancies were uncomplicated with normal vaginal deliveries. Her current pregnancy has progressed without complication, however at the last appointment she was informed that the baby was in breech position. For reasons unknown, Ms Spine has not had a preanesthetic evaluation before she presented herself and there has been no advance communication regarding her SCS [5]. Unfortunately, you do not have access to radiological imaging of the spinal stimulator due to electronic health records being disrupted by the cyberattack. However, on examination you observe the findings presented in Figure 1.

**Figure 1: Clinical examination of Ms Spine - Visual inspection revealed 1 horizontal scar and 2 midline vertical scars, corresponding to previous lead implantations for her stimulator.**

*[Nb. Figure 1 was taken from the clinical examination photos provided by Patel and Colleagues in reference 5, which reports an ‘Urgent Cesarean Section in a Patient with a Spinal Cord Stimulator: Implications for Surgery and Anesthesia’ [5].*

You must come up with a management plan for Ms Spine and consider the implications of her implanted technology for both herself and the baby.

**Case specific questions**

1. What are the risks of a compromised spinal cord stimulator during pregnancy, and how could a compromised device affect a fetus? In this particular case, what are the clinical implications of dealing with a closed-loop neuromodulation system for patient care?
2. How would you assess whether the medical device (i.e. the closed-loop spinal cord stimulator) is performing accordingly?
3. If you are concerned about the safety and performance of these devices, when and to whom do you communicate these concerns?
4. What measures and protocols are taken (by you or the hospital) to ensure that similar patient devices you may come in contact with are safe and are performing accordingly?
5. Do you have concerns about the reliability of AI-based or automated medical devices? If so, how does this impact your clinical decision-making and professional responsibility?

**General questions**

1. What challenges do clinicians face when dealing with the consequences of a cyberattack resulting in compromised digital records, devices, machinery, and/ or systems?
2. For outpatient care where remote services (i.e. cloud-based services) are increasingly used to monitor and tailor treatment regimes, how would compromises to these critical, yet externally managed, services affect patient care?
3. What challenges do clinicians face when using medical software and/ or (quasi)autonomous systems in patient care?

**References**

1. Ghafur, S., et al. ‘A Retrospective Impact Analysis of the WannaCry Cyberattack on the NHS’. Npj Digital Medicine, vol. 2, no. 1, Oct. 2019, pp. 1–7. <https://doi.org/10.1038/s41746-019-0161-6>.

2. Anderson T, Torreggiani W.C. ‘The Impact of the Cyberattack on Radiology Systems in Ireland’. Ir Med J; Vol 114; No. 5; P347. Available at: https://imj.ie/wp-content/uploads/2021/05/The-Impact-of-the-Cyberattack-on-Radiology-Systems-in-Ireland.pdf

3. Dameff, Christian, et al. ‘Cyber Disaster Medicine: A New Frontier for Emergency Medicine’. Annals of Emergency Medicine, vol. 75, no. 5, May 2020, pp. 642–47. ScienceDirect, <https://doi.org/10.1016/j.annemergmed.2019.11.011>.

4. Eichelberg, Marco, et al. ‘Cybersecurity Challenges for PACS and Medical Imaging’. Academic Radiology, vol. 27, no. 8, Aug. 2020, pp. 1126–39. ScienceDirect, <https://doi.org/10.1016/j.acra.2020.03.026>.

5. Patel, Suhas, et al. ‘Urgent Cesarean Section in a Patient with a Spinal Cord Stimulator: Implications for Surgery and Anesthesia’. The Ochsner Journal, vol. 14, no. 1, 2014, pp. 131–34. PubMed Central, <https://www.ncbi.nlm.nih.gov/pmc/articles/PMC3963044/>.

6. Vallejo, Ricardo, et al. ‘A New Direction for Closed-Loop Spinal Cord Stimulation: Combining Contemporary Therapy Paradigms with Evoked Compound Action Potential Sensing’. Journal of Pain Research, vol. 14, Dec. 2021, pp. 3909–18. PubMed Central, <https://doi.org/10.2147/JPR.S344568>.

7. Schultz, David M., et al. ‘Sensor-Driven Position-Adaptive Spinal Cord Stimulation for Chronic Pain’. Pain Physician, vol. 15, no. 1, 2012, pp. 1–12.

8. Zanos, Stavros. ‘Closed-Loop Neuromodulation in Physiological and Translational Research’. Cold Spring Harbor Perspectives in Medicine, vol. 9, no. 11, Nov. 2019, p. a034314. PubMed Central, <https://doi.org/10.1101/cshperspect.a034314>.

**Scenario 5: Patient care and autonomous ventilators**

**Scenario description**

You are a member of staff on the Intensive Care Unit (ICU) and have just arrived to take over from the night team. Overnight there was a cardiac arrest on the ward and the night team are debriefing.

The team are discussing **Mr Lungs** - a 60M admitted for septic shock who had been on a ventilator for three days. Overnight the following occurred:

- **0200am:** While ventilated with an FiO2 of 50%, a Vt of 430 mL, Mr Lungs SpO2 suddenly dropped from 97% to 85%. Nurse Cyber increased the FiO2 to 100% but the patient continued to have SpO2 at 82% [1].
- Dr Vent (the ICU doctor) assessed the patient and noticed that noticed that the ventilator displayed pressure and flow curves usually observed in pressure support mode (fixed pressure and decelerating flow), while it was still set in volume-controlled mode. Dr Vent reset the settings, confirming that the ventilator was still in volume-controlled mode.
- While doing this, the patient’s SpO2 dropped further, leading to hypoxic bradycardia and asystole. The patient was revived following the prompt withdrawal of the endotracheal tube, the provision of manual ventilation via bag and mask, and a brief cardiac massage. The patient was then placed back on the ventilator.

The team were now discussing what caused the event and Dr Vent (the ICU registrar) states that she believes that Nurse Cyber must have changed the ventilator mode from volume-controlled to pressure support. Nurse Cyber states that he did not do this, however he raises his concern that the ventilator might be faulty as he heard about a series of ventilators with closed-loop systems harming patients in France, and that their systems are vulnerable to cyberattacks [1-2].

While the staff are discussing these events, a nurse calls for assistance at the end of the ward as another patient - Mrs Chest, has developed respiratory distress and is desaturating to SpO2 70%. Mrs Chest (a 55F) was initially put on a ventilator due to respiratory failure relating to SARS-Cov-2 induced acute respiratory distress syndrome [1].

You attend to Mrs Chest and notice that this ventilator is also displaying inconsistent settings – the pressure and flow curves suggest that the ventilator is in pressure support mode, yet analysis of the settings states it is in volume-controlled mode.

You must form a management plan for Mrs Chest and for the other 10 patients on ICU who are all on the same type of ventilator.

**Case specific questions**

1. Which systems in Emergency and Intensive Care may be vulnerable to technical malfunctions and/or cyberattacks, and how might this manifest in patient illness?
2. What are the risks of closed-loop life support systems and medical systems based on Artificial Intelligence (AI) in acute medical settings?
3. If the patient had died, what are the appropriate post-mortem investigations and how should the cause of death be registered?
4. What measures and protocols are taken (by you or the hospital) to ensure the connected devices you are using are safe and performing accordingly?
5. Do you have concerns about the reliability of AI-based or automated medical devices?
6. If so, how does this impact your clinical decision-making and professional responsibility?

**General questions**

1. What challenges do clinicians face when dealing with the consequences of a cyberattack resulting in compromised digital records, devices, machinery, and/ or systems?
2. For outpatient care where remote services (i.e. cloud-based services) are increasingly used to monitor and tailor treatment regimes, how would compromises to these critical, yet externally managed, services affect patient care?
3. What challenges do clinicians face when using medical software and/ or (quasi)autonomous systems in patient care?

**References**

[1] Dufour, Nicolas, et al. ‘When a Ventilator Takes Autonomous Decisions without Seeking Approbation nor Warning Clinicians: A Case Series’. International Medical Case Reports Journal, vol. 13, 2020, pp. 521–29. PubMed, <https://doi.org/10.2147/IMCRJ.S266969>.

[2] Eliash, Carmel, et al. ‘SEC-C-U: The Security of Intensive Care Unit Medical Devices and Their Ecosystems’. IEEE Access, vol. 8, 2020, pp. 64193–224. IEEE Xplore, <https://doi.org/10.1109/ACCESS.2020.2984726>.

[3] Chalvignac, Philippe. Breathing Assistance Apparatus. CA2520326C, 22 Jan. 2013, <https://patents.google.com/patent/CA2520326C/en>.

[4] Sakiewicz, Paul G., et al. ‘Abnormal Electrical Stimulus of an Intra-Aortic Balloon Pump with Concurrent Support with Continuous Veno-Venous Hemodialysis’. ASAIO Journal, vol. 46, no. 1, Feb. 2000, p. 142. journals.lww.com, <https://journals.lww.com/asaiojournal/Fulltext/2000/01000/Abnormal_Electrical_Stimulus_of_an_Intra_Aortic.31.aspx>.

[5] Dameff, Christian J., et al. ‘Clinical Cybersecurity Training Through Novel High-Fidelity Simulations’. The Journal of Emergency Medicine, vol. 56, no. 2, Feb. 2019, pp. 233–38. ScienceDirect, <https://doi.org/10.1016/j.jemermed.2018.10.029>.

**Scenario 6: Seizure outbreaks in epilepsy management apps**

**Scenario description**

You work at a General Practice (GP) and have just seen your first patient - Miss Brain. Miss Brain is a 17F who tells you she had a relapse in her epilepsy this week, having experienced seizures in the evenings the last two nights. Both seizures came on when she was sat in bed at home, and states she was doing little at the time besides scrolling on her phone and checking the support forum of her epilepsy management app. Miss Brain shares that she is worried as two of her friends in her epilepsy support group have also had seizure relapses this week, without a clear cause.

Miss Brain was first diagnosed with Epilepsy 10 years ago and has been well-controlled and seizure-free for the past 3 years while managed on Sodium Valproate. The patient reports feeling otherwise well in herself, she has been taking her medications regularly, and has not experienced any other symptoms. Additionally, she denies starting any new medications or OTC drugs. She has been regularly using her epilepsy self-management app to record her mood, her medication adherence, and to chat with her support group in the app chat forum. Without indication of another cause, you question whether Miss Brain’s app use could have caused her symptoms. Miss Brain states that she does use the app regularly, but this has never caused her a problem in the past.

You are aware of the previous virtual assaults on epilepsy patients that have occurred online, including the harmful impact caused by hackers targeting epilepsy support forums with changes in screen brightness. You must come up with a management plan for Miss Brain and consider the wider population health implications of this case.

**Case specific questions**

1. How may cyberattacks on medical applications affect patients who suffer from photosensitive conditions, including migraine and epilepsy? What other patient groups may be at risk?
2. If you are concerned about the safety and performance of these medical applications, when, and to whom, do you communicate these concerns?
3. Who could you report this case to and where could you seek advice?
4. What measures and protocols are taken (by you or your practice) to ensure your concerns about patients interacting with potentially compromised medical apps are communicated and reported to the appropriate authorities/ entities?

**General questions**

1. What challenges do clinicians face when interacting with patients who use digital platforms and software as a medical device (SaMDs) to manage their conditions?
2. What challenges do clinicians face when dealing with the consequences of a cyberattack resulting in compromised digital records, medical devices (including apps), machinery, and/ or (automated) systems?
3. For outpatient care where remote services (i.e. cloud-based services) are increasingly used to monitor and tailor treatment regimes, how would compromises to these critical, yet externally managed, services affect patient care?

**References**

[1] Poulsen, Kevin. ‘Hackers Assault Epilepsy Patients via Computer’. Wired. [www.wired.com](http://www.wired.com), <https://www.wired.com/2008/03/hackers-assault-epilepsy-patients-via-computer/> Accessed 16 Jan. 2023

[2] Denning, Tamara, Yoky Matsuoka, and Tadayoshi Kohno. ‘Neurosecurity: Security and Privacy for Neural Devices’. Neurosurgical Focus 27, no. 1 (1 July 2009): E7. <https://doi.org/10.3171/2009.4.FOCUS0985>.

[3] Busby, Mattha. ‘Malicious Tweets Targeting Epilepsy Charity Trigger Seizures’. The Guardian, 15 May 2020. The Guardian,

<https://www.theguardian.com/society/2020/may/15/malicious-tweets-targeting-epilepsy-charity-trigger-seizures>.

[4] South, L., & Borkin, M. (2020, October 23). Ethical Considerations of Photosensitive Epilepsy in Mixed Reality. <https://doi.org/10.31219/osf.io/y32td>

[5] Alzamaman et al. (2021) ‘Self-Management Apps with People with Epilepsy: Systematic Analysis’. JMIR Mhealth Uhealth. 2021 May; 9(5): e22489. https://www.ncbi.nlm.nih.gov/pmc/articles/PMC8196364/
